# Estimates of SARS-CoV-2 Seroprevalence and Incidence of Primary SARS-CoV-2 Infections Among Blood Donors, by COVID-19 Vaccination Status — United States, April 2021–September 2022

**DOI:** 10.15585/mmwr.mm7222a3

**Published:** 2023-06-02

**Authors:** Jefferson M. Jones, Irene Molina Manrique, Mars S. Stone, Eduard Grebe, Paula Saa, Clara D. Germanio, Bryan R. Spencer, Edward Notari, Marjorie Bravo, Marion C. Lanteri, Valerie Green, Melissa Briggs-Hagen, Melissa M. Coughlin, Susan L. Stramer, Jean Opsomer, Michael P. Busch

**Affiliations:** ^1^National Center for Immunization and Respiratory Diseases, CDC; ^2^Westat, Rockville, Maryland; ^3^Vitalant Research Institute, San Francisco, California; ^4^American Red Cross, Washington, DC; ^5^Creative Testing Solutions, Tempe, Arizona.

Changes in testing behaviors and reporting requirements have hampered the ability to estimate the U.S. SARS-CoV-2 incidence (*1*). Hybrid immunity (immunity derived from both previous infection and vaccination) has been reported to provide better protection than that from infection or vaccination alone (*2*). To estimate the incidence of infection and the prevalence of infection- or vaccination-induced antibodies (or both), data from a nationwide, longitudinal cohort of blood donors were analyzed. During the second quarter of 2021 (April–June), an estimated 68.4% of persons aged ≥16 years had infection- or vaccination-induced SARS-CoV-2 antibodies, including 47.5% from vaccination alone, 12.0% from infection alone, and 8.9% from both. By the third quarter of 2022 (July–September), 96.4% had SARS-CoV-2 antibodies from previous infection or vaccination, including 22.6% from infection alone and 26.1% from vaccination alone; 47.7% had hybrid immunity. Prevalence of hybrid immunity was lowest among persons aged ≥65 years (36.9%), the group with the highest risk for severe disease if infected, and was highest among those aged 16–29 years (59.6%). Low prevalence of infection-induced and hybrid immunity among older adults reflects the success of public health infection prevention efforts while also highlighting the importance of older adults staying up to date with recommended COVID-19 vaccination, including at least 1 bivalent dose.*^,^^†^

Since July 2020, SARS-CoV-2 seroprevalence in the United States has been estimated by testing blood donations (*3*). CDC, in collaboration with Vitalant, American Red Cross, Creative Testing Solutions, and Westat, established a nationwide cohort of 142,758 blood donors in July 2021; the cohort included persons who had donated blood two or more times in the preceding year.^§^ All blood donations collected during April–June 2021 were tested for antibodies against the spike (S) and nucleocapsid (N) proteins. Beginning in 2022, up to one blood donation sample per donor was randomly selected each quarter and tested using the Ortho VITROS SARS-CoV-2 Quantitative S immunoglobulin G^¶^ and total N antibody** tests. Both SARS-CoV-2 infection and COVID-19 vaccination result in production of anti-S antibodies, whereas anti-N antibodies only result from infection. At each donation, blood donors were asked if they had received a COVID-19 vaccine. Using vaccination history and results of antibody testing, the prevalence of the U.S. population aged ≥16 years with vaccine-induced, infection-induced, or hybrid immunity was estimated for four 3-month periods (April–June 2021, January–March 2022, April–June 2022, and July–September 2022); in addition, the proportion of persons who transitioned from one immune status to another by quarter was estimated. Analysis was limited to 72,748 (51.0%) donors for whom it was possible to ascertain immune status during each period using their prior classification (e.g., previously infected or vaccinated), antibody testing results, and their vaccination status at the time of each donation.^††^ The sample data were weighted to account for selection into the study cohort, for nonresponse during the four analysis periods, and for demographic differences between the blood donor population and the overall U.S. population. The weights were obtained through a combination of stratification and raking, an iterative weighting adjustment procedure (*4*). Rates of infection among those previously uninfected were estimated for each period by determining the percentage of anti-N–negative persons seroconverting to anti-N–positive from one 3-month period included in the study to the next. Estimates were stratified by age group (16–29, 30–49, 50–64, and ≥65 years) and race and ethnicity^§§^ (Asian, Black or African American [Black], White, Hispanic or Latino [Hispanic], and other). SAS (version 9.4; SAS Institute) was used to compute the final weights, and R (version 4.2.1; R Foundation) was used to calculate all the estimates and create the plots.^¶¶^ Seroprevalence and infection rates were estimated as weighted means and compared by demographic group and vaccination status using two-sided t-tests with a significance level of α = 0.05. This activity was reviewed by CDC and conducted consistent with applicable federal law and CDC policy.***

During the first quarter examined (April–June 2021), an estimated 68.4% (95% CI = 67.8%–68.9%) of persons aged ≥16 years had SARS-CoV-2 antibodies from previous infection or vaccination, including 47.5% (95% CI = 46.0%–49.0%) from vaccination alone, 12.0% (95% CI = 10.8%–13.5%) from infection alone, and 8.9% (95% CI = 8.7%–9.2%) from both (Figure 1) (Supplementary Figure 1, https://stacks.cdc.gov/view/cdc/128630). During January–March 2022, 93.5% (95% CI = 93.1%–93.9%) of persons aged ≥16 years had antibodies from previous infection or vaccination, including 39.0% (95% CI = 37.4%–40.7%) from vaccination alone, 20.5% (95% CI = 19.2%–22.2%) from infection alone, and 34.1% (95% CI = 32.4%–35.8%) from both. During July–September 2022, 96.4% (95% CI = 96.1%–96.7%) of persons had antibodies from previous infection or vaccination, including 26.1% (95% CI = 25.4%–26.9%) with vaccine-induced immunity alone, 22.6% (95% CI = 21.2%–24.1%) with infection-induced immunity alone, and 47.7% (95% CI = 44.8%–51.2%) with hybrid immunity. During July–September 2022, the prevalence of infection-induced immunity was 85.7% (95% CI = 79.8%–90.2%) among unvaccinated persons and 64.3% (95% CI = 61.9%–66.7%) among vaccinated persons.

**FIGURE 1:**
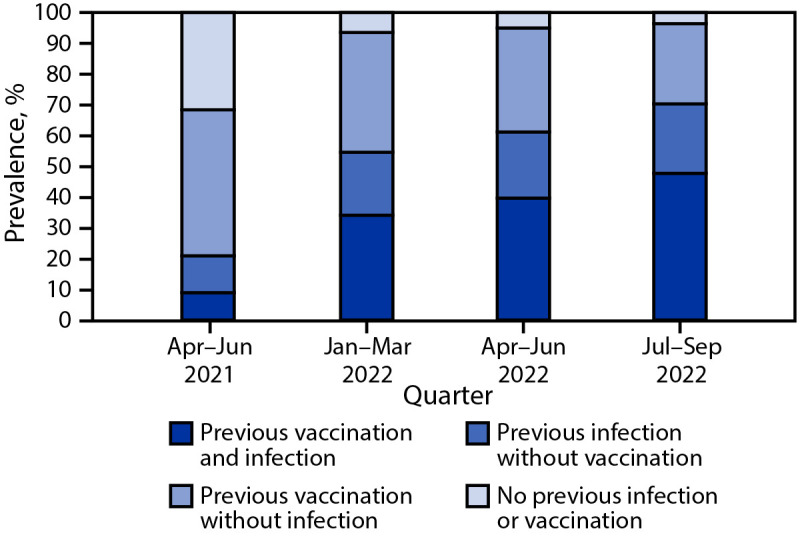
Prevalences of vaccine-induced, infection-induced, and hybrid* immunity^†^ against SARS-CoV-2 among blood donors aged ≥16 years — United States, April 2021–September 2022 * Immunity derived from a combination of vaccination and infection. **^†^** Ascertained by the presence of anti-spike antibodies (present in both COVID-19–vaccinated and SARS-CoV-2–infected persons) and anti-nucleocapsid antibodies (present only in previously infected persons) and self-reported history of vaccination.

During July–September 2022, the lowest prevalence of hybrid immunity, 36.9% (95% CI = 35.8%–38.1%), was observed in persons aged ≥65 years, and the highest, 59.6% (95% CI = 56.7%–62.3%), in adolescents and young adults aged 16–29 years (Figure 2) (Supplementary Figure 2, https://stacks.cdc.gov/view/cdc/128679). During all periods, higher prevalences of hybrid immunity were observed among Black and Hispanic populations than among White and Asian populations (Supplementary Figure 3, https://stacks.cdc.gov/view/cdc/128680).

**FIGURE 2:**
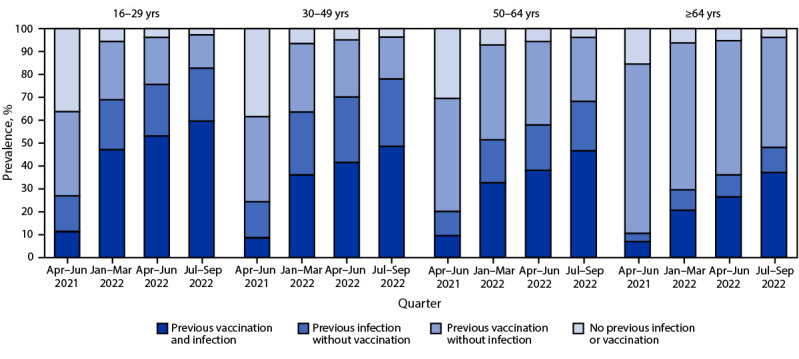
Prevalences of vaccine-induced, infection-induced, and hybrid* immunity^†^ against SARS-CoV-2 among blood donors aged ≥16 years, by age group — United States, April 2021–September 2022 * Immunity derived from a combination of vaccination and infection. **^†^** Ascertained by the presence of anti-spike antibodies (present in both COVID-19–vaccinated and SARS-CoV-2–infected persons) and anti-nucleocapsid antibodies (present only in previously infected persons) and self-reported history of vaccination.

Among persons with no previous infection, the incidence of first infections during the study period (i.e., conversion from anti-N–negative to anti-N–positive) was higher among unvaccinated persons (Table). From April–June 2021 through January–March 2022, the incidence of first SARS-CoV-2 infections among unvaccinated persons was 67.0%, compared with 26.3% among vaccinated persons (p<0.05). From January–March 2022 through April–June 2022, the incidence among unvaccinated persons was 21.7% and was 13.3% among vaccinated persons. Between April–June 2022 and July–September 2022, the incidence among unvaccinated persons was 28.3%, compared with 22.9% among vaccinated persons (p<0.05). Incidence of first SARS-CoV-2 infections was higher among younger than among older persons and was lower among Asian persons than among other racial and ethnic populations, but the differences among groups narrowed over time.

**TABLE:** Estimated percentage* of persons infected with SARS-CoV-2 for the first time among blood donors, by analysis quarter, sociodemographic characteristics, and vaccination status — United States, April 2021**–**September 2022

Characteristic	Period, % (95% CI)		
	Apr–Jun 2021 to Jan–Mar 2022	Jan–Mar 2022 to Apr–Jun 2022	Apr–Jun 2022 to Jul–Sep 2022
**Overall**			
**Total**	**42.5 (41.8–43.3)**	**14.5 (13.7–15.3)**	**23.6 (22.8–24.5)**
Unvaccinated	67.0 (65.6–68.4)^†^	21.7 (19.1–24.4)^†^	28.3 (25.5–31.3)^†^
Vaccinated	26.3 (25.4–27.1)	13.3 (12.4–14.1)	22.9 (22.1–23.8)
**Age group, yrs**			
**16–29**			
**Total**	**57.4 (54.8–59.9)**	**21.8 (18.6–25.4)**	**29.3 (25.8–33.0)**
Unvaccinated	73.8 (69.5–77.7)	31.5 (21.5–43.7)	29.5 (18.1–44.2)
Vaccinated	41.2 (38.1–44.4)	19.7 (16.6–23.3)	29.2 (25.6–33.1)
**30–49**			
**Total**	**51.8 (50.4–53.3)**	**18.0 (16.1–20.0)**	**26.8 (24.9–28.8)**
Unvaccinated	70.6 (68.5–72.5)	23.0 (17.9–28.9)	25.6 (21.3–30.4)
Vaccinated	32.5 (30.6–34.4)	16.9 (15.0–18.9)	27.0 (25.0–29.2)
**50–64**			
**Total**	**38.9 (37.3–40.5)**	**13.2 (12.0–14.6)**	**24.1 (22.4–25.9)**
Unvaccinated	61.5 (58.5–64.4)	19.8 (16.0–24.2)	32.0 (27.5–36.9)
Vaccinated	24.6 (23.1–26.3)	12.1 (10.8–13.6)	22.9 (21.2–24.7)
**≥65**			
**Total**	**21.0 (20.0–22.2)**	**9.2 (8.4–10.0)**	**18.5 (17.4–19.7)**
Unvaccinated	49.6 (46.3–52.9)	13.7 (11.3–16.5)	27.0 (22.8–31.6)
Vaccinated	15.0 (13.9–16.2)	8.7 (7.9–9.6)	17.8 (16.6–19.0)
**Race and ethnicity^§^**			
**Asian**			
**Total**	**29.1 (26.0–32.3)**	**8.9 (6.6–11.8)**	**23.2 (19.5–27.5)**
Unvaccinated	53.1 (40.7–65.0)	6.3 (1.9–18.8)	22.1 (8.3–47.0)
Vaccinated	24.7 (21.7–27.9)	9.0 (6.7–12.1)	23.3 (19.5–27.5)
**Black or African American**			
**Total**	**42.4 (37.8–47.2)**	**12.9 (9.5–17.4)**	**23.7 (19.4–28.6)**
Unvaccinated	71.6 (61.0–80.3)	14.8 (3.0–49.5)	21.7 (5.0–59.3)
Vaccinated	30.1 (25.9–34.6)	12.8 (9.3–17.3)	23.9 (19.7–28.6)
**White**			
**Total**	**43.2 (42.4–43.9)**	**15.3 (14.6–16.1)**	**23.5 (22.8–24.3)**
Unvaccinated	67.4 (66.0–68.8)	22.7 (20.1–25.6)	29.5 (26.6–32.5)
Vaccinated	23.5 (22.9–24.1)	13.8 (13.1–14.6)	22.5 (21.7–23.3)
**Hispanic or Latino**			
**Total**	**45.5 (42.9–48.2)**	**14.0 (11.8–16.4)**	**23.4 (20.9–26.1)**
Unvaccinated	64.6 (59.9–69.1)	17.9 (12.2–25.4)	27.2 (18.7–37.9)
Vaccinated	34.5 (31.6–37.5)	13.3 (11.0–16.1)	22.8 (20.3–25.6)
**Other and multiple races^¶^**			
**Total**	**43.4 (38.3–48.7)**	**18.1 (12.7–25.2)**	**27.3 (21.9–33.6)**
Unvaccinated	65.7 (56.5–73.9)	33.4 (15.1–58.7)	21.6 (9.9–40.8)
Vaccinated	28.1 (22.8–34.0)	14.7 (10.4–20.4)	28.3 (22.3–35.2)

* Percentage of uninfected persons (anti-nucleocapsid–negative in the previous 3-month period) seroconverting to anti-nucleocapsid–positive. If both vaccination and infection occurred between donations included in the study, the order could not be determined, and some unvaccinated donors might have been vaccinated before infection and thus misclassified.

^†^ If donors who transitioned from no antibodies to hybrid immunity between April–June 2021 and January–March 2022 were excluded, an estimated 55.5% (95% CI = 53.9%–57.1%) of unvaccinated donors were infected. For other periods, exclusion did not substantially change results. Between January–March and April–June 2022, 0.4% of persons shifted from no antibodies to hybrid immunity. Between April–June and July–September 2022, 0.3% of persons shifted from no antibodies to hybrid immunity.

^§^ Persons of Hispanic or Latino (Hispanic) origin might be of any race but are categorized as Hispanic; all racial groups are non-Hispanic.

^¶^ Includes American Indian or Alaska Native and non-Hispanic persons of other races.

## Discussion

Both infection-induced and hybrid immunity increased during the study period. By the third quarter of 2022, approximately two thirds of persons aged ≥16 years had been infected with SARS-CoV-2 and one half of all persons had hybrid immunity. Compared with vaccine effectiveness against any infection and against severe disease or hospitalization, the effectiveness of hybrid immunity against these outcomes has been shown to be higher and wane more slowly (*2*). This increase in seroprevalence, including hybrid immunity, is likely contributing to lower rates of severe disease and death from COVID-19 in 2022–2023 than during the early pandemic.^†††^ The prevalence of hybrid immunity is lowest in adults aged ≥65 years, likely due to higher vaccination coverage and earlier availability of COVID-19 vaccines for this age group, as well as to higher prevalences of behavioral practices to avoid infection (*5*). However, lower prevalences of infection-induced and hybrid immunity could further increase the risk for severe disease in this group, highlighting the importance for adults aged ≥65 years to stay up to date with COVID-19 vaccination and have easy access to antiviral medications.

COVID-19 vaccine efficacy studies have reported reduced effectiveness against SARS-CoV-2 infection during the Omicron-predominant period compared with earlier periods and have shown that protection against infection wanes more rapidly than does protection against severe disease (*6*,*7*). In this study, unvaccinated persons had higher rates of infection (as evidenced by N antibody seroconversion) than did vaccinated persons, indicating that vaccination provides some protection against infection. The differences in incidence could also be due to systematic differences between vaccinated and unvaccinated persons in terms of the prevalence of practicing prevention behaviors such as masking and physical distancing. The relative difference in infection rates narrowed during the most recent months, possibly because of waning of vaccine-induced protection against infection in the setting of increased time after vaccination or immune evasion by the SARS-CoV-2 Omicron variant. The narrowing of difference in infection rates might also be attributable to increasing similarities in behavior among vaccinated and unvaccinated persons during late 2022 (*8*).

The findings in this report are subject to at least six limitations. First, although COVID-19 booster vaccine doses and reinfections can strengthen immunity (*9*,*10*), this analysis did not account for these effects because blood donor vaccination history did not include the number of doses received, and data on reinfections were not captured. Second, immunity wanes over time, but time since vaccination or infection was not included in the analysis (*2*). Third, vaccination status was self-reported, potentially leading to misclassification. Fourth, although the results were adjusted based on differences in blood donor and general population demographics, estimates from blood donors might not be representative of the general population; thus, these results might not be generalizable. Fifth, vaccinated and unvaccinated persons might differ in other ways not captured by this analysis (*8*), nor can causality be inferred from the results on relative infection incidence. Finally, if both vaccination and infection occurred between blood donations included in the study, the order of occurrence could not be determined, and some unvaccinated donors might have been vaccinated before infection and thus misclassified; in 2022, this was uncommon and occurred in <0.5% of donors during any 3-month period.

This report found that the incidence of first-time SARS-CoV-2 infection was lower among persons who had received COVID-19 vaccine than among unvaccinated persons and that infection-induced and hybrid immunity have increased but remain lowest in adults aged ≥65 years. These adults have consistently had a higher risk for severe disease compared with younger age groups, underscoring the importance of older adults staying up to date with recommended COVID-19 vaccination, including at least 1 bivalent dose.

**Summary**

**What is already known about this topic?**

SARS-CoV-2 hybrid immunity (immunity derived from both previous infection and vaccination) has been reported to provide better protection than that from infection or vaccination alone.

**What is added by this report?**

By the third quarter of 2022, an estimated 96.4% of persons aged ≥16 years in a longitudinal blood donor cohort had SARS-CoV-2 antibodies from previous infection or vaccination, including 22.6% from infection alone and 26.1% from vaccination alone; 47.7% had hybrid immunity. Hybrid immunity prevalence was lowest among adults aged ≥65 years.

**What are the implications for public health practice?**

Low prevalence of infection-induced and hybrid immunity among older adults, who are at increased risk for severe disease if infected, reflects the success of public health infection prevention efforts while also highlighting the importance of this group staying up to date with recommended COVID-19 vaccination, including at least 1 bivalent dose.

## Footnotes

*
https://www.cdc.gov/coronavirus/2019-ncov/your-health/risks-getting-very-sick.html

†
https://www.cdc.gov/coronavirus/2019-ncov/vaccines/index.html

§ Blood donors who donated at least twice during the year before July 2021 were included in the cohort, because they might represent persons who were more likely to donate frequently. Among donors who donated more than once during a quarter, one sample was selected at random for testing.

¶
https://www.fda.gov/media/150675/download

††
https://www.cdc.gov/coronavirus/2019-ncov/hcp/testing/antibody-tests-guidelines.html

§§ Persons of Hispanic origin might be of any race but are categorized as Hispanic; all racial groups are non-Hispanic.

¶¶ Jackknife replication was used to compute replicate weights. Weights were adjusted for nonresponse using adjustment cells created by age category, vaccination and previous infection status, and blood collection organization (Vitalant or American Red Cross). Raking was used to further adjust the weights to account for demographic differences between the blood donor population and U.S. population. The demographic variables used for raking were sex (female and male), age group (16–24, 25–34, 35–44, 45–54, 55–64, and ≥65 years), and race and ethnicity (Asian, Black, White, Hispanic, and other).

*** 45 C.F.R. part 46, 21 C.F.R. part 56; 42 U.S.C. Sect. 241(d); 5 U.S.C. Sect. 552a; 44 U.S.C. Sect. 3501 et seq.

††† https://covid.cdc.gov/covid-data-tracker/#datatracker-home (Accessed May 25, 2023).
